# Variation in harms and benefits of prostate‐specific antigen screening for prostate cancer by socio‐clinical risk factors: A rapid review

**DOI:** 10.1002/bco2.326

**Published:** 2024-02-09

**Authors:** Abel Tesfai, Natalia Norori, Thomas A. Harding, Yui Hang Wong, Matthew David Hobbs

**Affiliations:** ^1^ Prostate Cancer UK London UK

**Keywords:** age, ethnicity, family history, mortality, overdiagnosis, risk factors, screening

## Abstract

**Objective:**

To analyse the latest evidence on the relative harms and benefits of screening and diagnostic pathways with close examination of (i) men aged 50 years or older, (ii) men whose ethnicity places them at higher risk and (iii) men with a family history.

**Methods:**

We conducted a literature search using PubMed and Cochrane Central Register of Controlled Trials (CENTRAL) databases and other sources, from January 1990 to 25 January 2023. Two independent reviewers selected for randomised controlled trials (RCTs) and cohort studies which met our inclusion criteria.

**Results:**

Twenty‐eight articles were selected, from six trials, including the Göteborg trial—reported separately from European Randomised Study of Screening for Prostate Cancer (ERSPC). Prostate‐specific antigen (PSA)‐based screening led to the increased detection of low‐grade cancer and reduction of advanced/metastatic disease but had contradictory effects on prostate cancer (PCa)‐specific mortality (no difference or reduced), possibly due to issues of contamination or compliance. Screening men from a relatively young age (50–55) reduced risk of PCa‐specific mortality in a subanalysis of an 18‐year follow‐up study and in a 17‐year cohort study from the main Göteborg trial. Moreover, one Prostate, Lung, Colorectal and Ovarian (PLCO) Cancer Screening Trial analysis reported a trend of reduced risk of PCa‐specific mortality for men with a family history who were screened. [Correction added on 05 March 2024, after first online publication: “Cancer Screening Trial” has been added to the preceding sentence.] However, we did not find relevant studies for ethnicity.

**Conclusion:**

Under current UK practice, the choice to conduct a PSA test relies on a shared decision‐making approach guided by known risk factors. However, we found there was a lack of strong evidence on the harms and benefits of PSA screening by socio‐clinical risk factors and suggest further research is required to understand the long‐term impact of screening on high‐risk populations in the current diagnostic setting.

## 1 INTRODUCTION

Prostate cancer (PCa) is the second most common cancer in men worldwide and the fifth leading cause of death.^1^ In the United Kingdom, PCa is the most diagnosed cancer in men, affecting an average of 52 000 men every year. PCa is also associated with the death of 12 000 men every year.^2^ Organised screening of asymptomatic men uses prostate‐specific antigen (PSA) testing to detect cancer in its early stages and reduce PCa‐related morbidity and mortality. Debates on the harms and benefits of screening have focused on findings from three key multi‐arm randomised controlled trials (RCTs) and have also been informed by knowledge of the PSA tests' low sensitivity and the risk of overdiagnosis. The European Randomised Study of Screening for Prostate Cancer (ERSPC) reported 21% relative risk reduction for PCa mortality after a median follow‐up of 13 years, although this varied across centres.^3^ However, these findings were not replicated in the Prostate, Lung, Colorectal and Ovarian (PLCO) Cancer Screening Trial and the Cluster Randomised Trial of PSA (CAP) testing for PCa.^4^ [Correction added on 05 March 2024, after first online publication: “Cancer Screening Trial” has been added to the preceding sentence.] Furthermore, organised PCa screening has been associated with harms, including overdiagnosis and overtreatment of clinically insignificant PCa.^5^, ^6^, ^7^, ^8^

In the United Kingdom, patient referral to the diagnostic pathway is based on clinical judgement in primary care, with most patients presenting with symptoms such as lower urinary tract symptoms, which may result from benign conditions.^9^ PSA testing in the United Kingdom has also been associated with a median lead time bias of 7 years.^5^ Despite this, there have been reports of PSA testing as high as 44%, in a 19‐year follow‐up study of men aged 40–75,^10^ with another study also showing regional differences in testing,^11^ indicating considerable uptake of opportunistic PSA testing in the absence of a nationwide screening programme. In Europe, there have been calls to consider a risk‐stratified approach to early diagnosis of PCa.^12^, ^13^ Of relevance, Lithuania implemented a risk‐based approach to population screening, which targeted (i) men aged 50–74 and (ii) men aged 45–49 with family history, and used a PSA referral threshold of ≥3 ng/mL. More specifically, an analysis of this programme's multiple rounds of screening, that is, men monitored due to a PSA of ≤3 ng/mL, found greater detection of organ‐confined (localised) PCa.^14^

In this review, we define men at risk as men aged 50 years or older. Men at higher risk were defined as men over 45, who have a first‐degree relative diagnosed with prostate, breast, or ovarian cancer, or are of Black ethnicity. Multiple studies helped inform our inclusion criteria. For example, recent randomised trial data have reported a reduction in the risk of dying from PCa when starting screening at 55 rather than at 60 years of age.^15^ However, men may also die from competing comorbidities associated with old age. Moreover, men with a family history, namely, men with first‐degree relatives with PCa, breast or ovarian cancer, have been associated with an increased risk of PCa.^16^ Reports have also suggested that the early onset of PCa in a sibling may increase the risk of PCa diagnosis.^17^ Furthermore, Black men have been reported to have a 1 in 4 lifetime risk of being diagnosed with PCa and a 1 in 12 risk of dying from it, which is double the risk to White men, which were found to be 1 in 8 and 1 in 24, respectively.^18^ In addition, a prospective study among Black men in the United States found that baseline PSA testing at midlife (40–64) can effectively predict total and aggressive cancer.^19^ However, PSA levels have also previously been reported to vary across different ethnicities.^20^ To our knowledge, no clinical trial has explored and reported whether Black men benefit more from screening compared with other men. Therefore, in this review, we sought to (i) analyse the latest evidence on the relative harms and benefits of screening and diagnostic pathways (as comparator) and (ii) identify how this applies to men at risk and higher risk of PCa.

## 2 METHODS

### 2.1 Search strategy

We performed a search using PubMed and Cochrane Central Register of Controlled Trials databases and other sources including web searches for guidelines (European Association of Urology) and trial search portals (Tables S1–S4).^21^ The inclusion criteria were as follows: (i) men over 45, men with a first‐degree relative with prostate, breast or ovarian cancer, Black men (population); (ii) PSA‐based screening with or without MRI (intervention); (iii) current standard of care, where 'no screening' was used as a comparator; and (iv) harms and benefits of PCa screening (used to evaluate outcome). Studies were RCT and cohort studies, published between 01 January 1990 and 25 January 2023.

### 2.2 Study selection

Retrieved articles were reviewed using the systematic review tool Rayyan.^22^ Two independent reviewers subsequently conducted a pilot test and performed a blinded selection process involving 215 abstracts and 88 full texts. Conflict between reviewers were either resolved between reviewers or were reconciled by a third team member following discussions. Some key decisions made by reviewers were to include (i) follow‐up trials, (ii) articles examining contamination within the ERSPC study and exclude microsimulation studies on the basis that they were not true intervention trials. This selection process led to data extraction of 28 articles.^3^, ^4^, ^23^, ^24^, ^25^, ^26^, ^27^, ^28^, ^29^, ^30^, ^31^, ^32^, ^33^, ^34^, ^35^, ^36^, ^37^, ^38^, ^39^, ^40^, ^41^, ^42^, ^43^, ^44^, ^45^, ^46^, ^47^, ^48^ Other studies were used to contextualise and update findings where relevant.^15^, ^49^, ^50^

### 2.3 Data extraction

Data extraction was conducted independently by three reviewers using a tested and standardised form, according to Cochrane templates for data extraction. A single reviewer also re‐examined 30% of papers that were extracted by the other two reviewers to check for both completeness and correctness of data.

### 2.4 Risk of bias assessment

We closely adhered to Cochrane's risk of bias assessment guideline for rapid reviews. A single reviewer conducted risk of bias assessment, with a second reviewer re‐examining 20% of articles. Risk of bias assessment was conducted by analysing the main study report, supplementary papers and available protocols.

Risk of bias was assessed using the following tools: (i) RoB 2 (version 2019; 25 articles) for RCTs, (ii) RoB 2 for cluster randomised trials (version 2021; one article) and Robins‐I (version 2016; two articles) for non‐randomised trial studies, across defined domains, described in Table S5.^51^

Overall risk of bias were judged as low risk of bias, high risk of bias or some concerns, for RoB 2 and RoB 2 for cluster randomised trials while for Robins‐I, judgements were summarised as low risk of bias, serious risk of bias, critical risk of bias or moderate risk of bias concerns; however, for simplicity, judgements for Robins‐I were consolidated to reflect the lack of critical risk of bias found in studies. Summary of risk of bias assessment was visualised using robvis tool. Importantly, no exclusions were made based on assessments.

## 3 RESULTS

### 3.1 Search results and study characteristics

**FIGURE 1:**
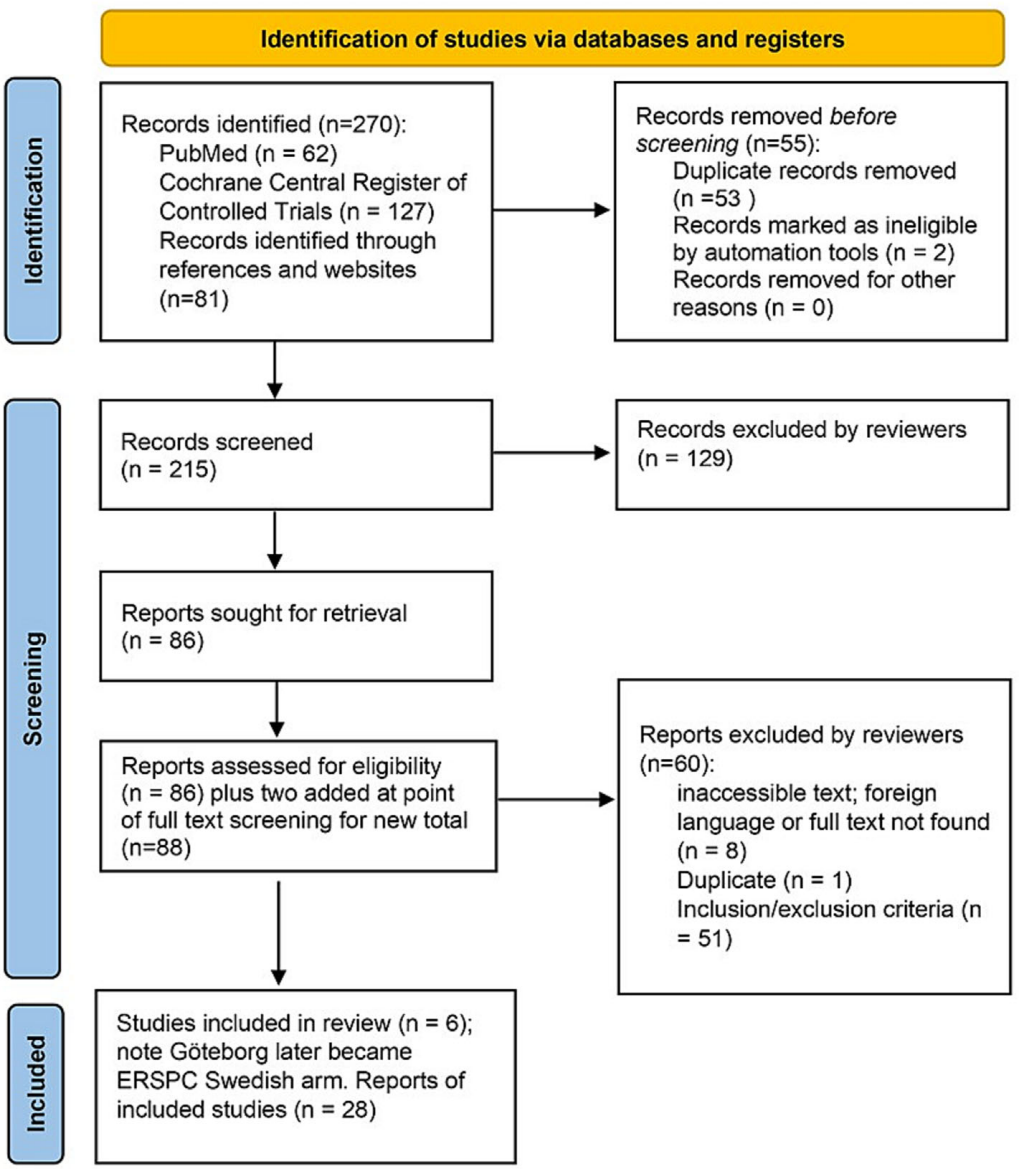
PRISMA flow diagram. Searches were conducted using PubMed, Cochrane Central Register of Controlled Trials and records identified through references (citations) and websites.

A total of 28 articles sourced from six trials were included in the analysis (Figure 1). Most articles were linked to official ERSPC follow‐up studies and individual ERSPC trial arm studies (*n* = 20). Table 1 provides an overview of the key features of each study. Among these, four were randomised control trials with appropriate analysis reported (namely, ERSPC, Göteborg, CAP and PLCO), one was a quasi‐randomised control trial (Norrköping) while another RCT study had an unclear randomisation process (Quebec).^52^

**TABLE 1:** Study characteristics.

Trial study articles and number	Study design and setting	Latest median follow‐up (years)	Inclusion criteria	Number of men included in analysis (intervention/Control)	Intervention/comparator	Screening interval	Key outcomes
ERSPC core; ISRCTN49127736^3^	Multi‐centre RCT Countries: The Netherlands, Sweden, Finland, Belgium, France, Spain, Italy and Switzerland	16	Men aged 55–69 years, no previous diagnosis of prostate cancer	72 891/89 352	PSA test; if PSA ≥ 3 ng/mL with further testing including DRE, standard TRUS biopsy and laterally directed sextant biopsies/no PSA‐based screening	2–4 years	PCa‐specific mortality, overall mortality, PCa incidence, PCa stage, survival, contamination (Rotterdam), effect of family history (Switzerland)
Göteborg; ISRCTN54449243^40^	RCT (part of ERSPC), Country: Sweden—Göteborg	22	Men aged 50–64 at time of recruitment with a median upper age limit of 69 set for subsequent screening rounds	9945/9949	PSA screening, with further testing if PSA ≥ 3–4 ng/mL/no PSA‐based screening	2 years	PCa‐specific mortality, overall mortality, PCa incidence, PCa stage, survival, effect of age and socio‐economic variables, side effects from treatment, contamination
CAP; ISRCTN92187251^4^	Multi‐centre cluster RCT Country: UK	10	Men aged 50–69 years, no previous diagnosis of prostate cancer	189 386/219 439	Prostate biopsy if PSA ≥ 3 ng/mL/no screening	1 time only	PCa‐specific mortality, overall mortality, PCa incidence, PCa stage, quality of life
PLCO; NCT00002540^31^	Multi‐centre RCT Country: USA	16.9	Men aged 55–74 years, no previous diagnosis of prostate cancer	38 340/38 343	PSA test and DRE. Further testing if PSA ≥ 3 ng/mL/no PSA‐based screening	Annual PSA testing for 6 years and annual DRE for 4 years	PCa‐specific mortality, overall mortality, PCa incidence, PCa grade and stage, contamination, effect of family history
Norrköping; ISRCTN06342431^47^	Pseudo‐RCT Country: Sweden	20	Men aged 50–69 years, no previous diagnosis of prostate cancer	1494/7532	DRE only for the first two screening rounds but PSA and DRE for remaining rounds. Prostate biopsy if suspicious DRE or PSA ≥ 4 ng/mL/no PSA‐based screening	3 years	PCa‐specific mortality, overall mortality, PCa incidence, PCa stage and grade
Quebec; no trial number^43^	RCT Country: Canada—Quebec (results not reported for RCT)	8	Men aged 40–80 years, no previous diagnosis of prostate cancer	31 300/15 432	PSA test and DRE, with prostate biopsy if PSA ≥ 3 ng/mL/no PSA‐based screening	Annual PSA testing	PCa‐specific mortality, PCa incidence, PCa stage

*Note*: Study details are sourced from latest relevant articles. In the ERSPC study article a median follow‐up of 15.5 years is quoted with a maximum follow‐up of 16 years reported for multiple centres. This has been simplified in the table, to describe the median follow‐up as 16 years. [Correction added on 05 March 2024, after first online publication: In the footnote to Table 1, the Note has been revised.]

Abbreviations: CAP, Cluster Randomised Trial of PSA Testing for Prostate Cancer; DRE, digital rectal examination; ERSPC, European Randomised Study of Screening for Prostate Cancer; PCa, prostate cancer; PLCO, Prostate, Lung, Colorectal and Ovarian Cancer Screening Trial; ProtecT, Prostate Testing for Cancer and Treatment; PSA, prostate‐specific antigen; TRUS, transrectal ultrasonography; RCT, randomised controlled trial.

[Correction added on 05 March 2024, after first online publication: Reference citation to “PLCO; NCT00002540” has been corrected.]

The ERSPC study is one of the largest RCTs to date, assessing the effectiveness of PSA‐based screening in reducing PCa‐specific mortality. The trial took place in eight centres across Europe. In this review, we report results for the core group of men randomised, with a total of 162 241 men aged 55–69 from seven of those centres, excluding France. The Göteborg trial, which started in 1995 and randomised 19 894 men, served as the Swedish component of ERSPC starting from 1996. Therefore, its results are incorporated within the primary reports of the ERSPC trial. The CAP trial included over 400 000 men across 573 centres.

The PLCO trial took place in the United States and included a total of 76 683 men in the analysis. The Norrköping study was a quasi‐randomised control trial that included 9026 men. One thousand four hundred ninety‐four men were allocated to the screening arm and the rest to the control arm. The Quebec trial had an ambiguous randomisation process and presented observational outcomes by comparing screened and unscreened men, instead of by trial arm, and thus should be interpreted with caution.

### 3.2 Quality assessment

All reported studies were included in the risk of bias assessment. Of the 28 randomised study articles assessed using the tool, six were judged to be low risk. These were predominately linked to Göteborg section of ERSPC trial. In the remaining randomised studies, some concerns were identified in six study articles while 16 study articles were judged as being at high risk of bias. Study articles judged to be of high risk were predominantly from multi‐arm ERSPC trial due to order of consent and randomisation in Netherlands, Switzerland, Belgium and Spain sections of ERSPC study, which can potentially impact allocation concealment, but also included PLCO, known to have severe contamination, and CAP which had a flawed allocation of GP practices. Issues were also identified in four out of five risk of bias domains for the Quebec trial, with some concerns in Domains 1 and 5 and high risk in Domains 2 and 4, due to issues of allocation concealment, deviations from intended interventions and bias in measurement and reporting of outcomes.

**FIGURE 2:**
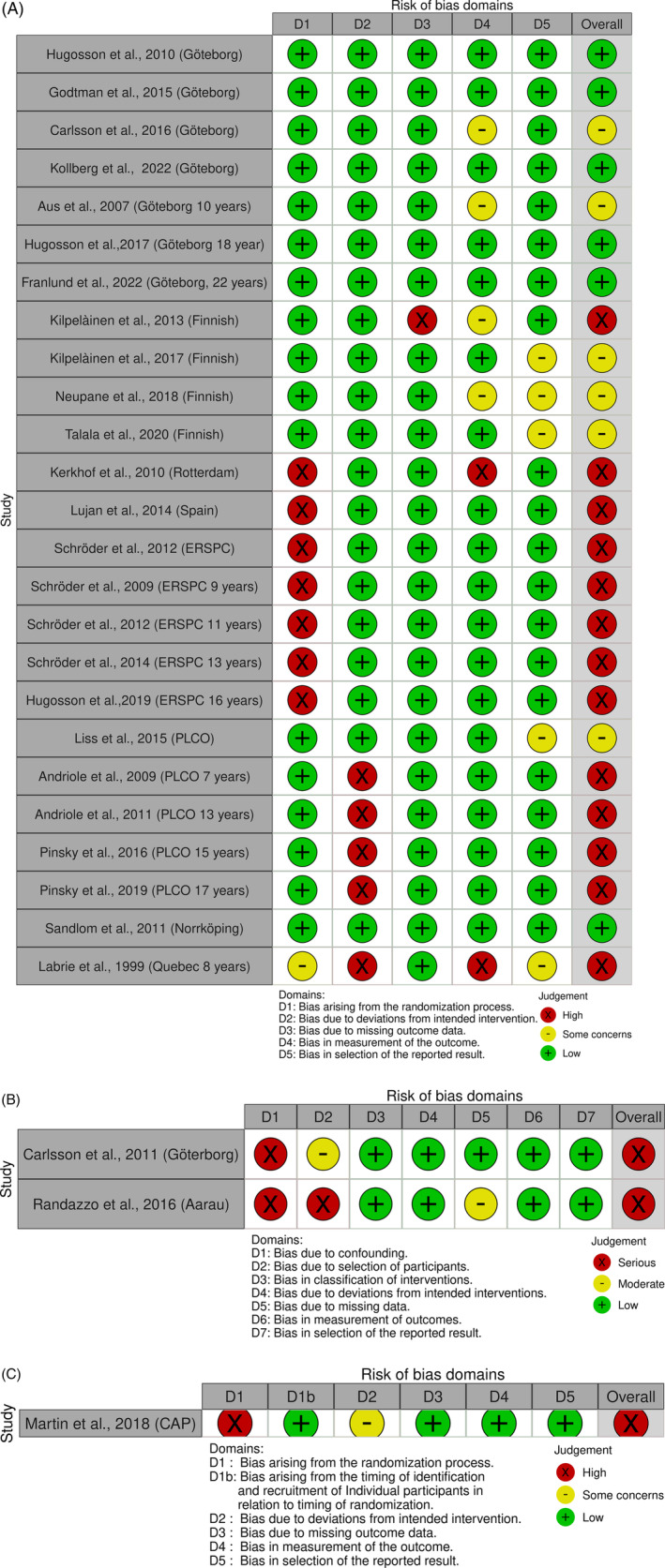
(A–C) Risk of bias summaries for randomised trials; RoB 2 (A), non‐randomised trials; Robins‐I (B) and cluster randomised trials; RoB 2 for cluster randomised trials (C). Study articles from main trials contain follow‐up years in description. CAP, Cluster Randomised Trial of PSA Testing for Prostate Cancer; ERSPC, European Randomised Study of Screening for Prostate Cancer; PLCO, Prostate, Lung, Colorectal and Ovarian Cancer Screening rial; ProtecT, Prostate Testing for Cancer and Treatment.

Two non‐randomised and one cluster randomised trial study articles were assessed using two tools: Robins‐I and RoB 2 for cluster randomised trial, respectively. These trial study articles were subsequently found to be at serious risk of bias. This was primarily due to a lack of control for baseline confounding or possible selection bias. Figure 2 summarises the risk of bias assessment for each study article.

### 3.3 PCa‐specific mortality

A summary of the key outcomes of each study are presented below and in Tables 2 and 3. According to findings from the ERSPC trial, repeated PSA PCa screening, with an average screening intensity of 4 years and mean screening round per man of 1.9, for PCa led to a relative risk reduction of 20% in PCa‐specific mortality over a follow‐up period of 16 years. With increased duration of follow‐up, there was a decrease in the number needed to invite (NNI) and the number needed to diagnose (NND) to prevent one PCa death; from 742 invited men and 26 diagnosed to prevent one PCa death at 13 years of follow‐up to 570 invited men and 18 diagnosed after 16 years of follow‐up. The Göteborg trial reported results after 22 years of follow‐up and found a 30% reduction in PCa‐specific mortality. The NNI decreased from 243 to 217, and the NND decreased from 11 to 9 at 18 and 22 years of follow‐up, respectively.

**TABLE 2:** Key outcomes.

Outcomes	ERSPC, 16‐year median follow‐up^3^	Göteborg, 22 (and 18)‐year median follow‐up^29^, ^40^	CAP, 10‐year median follow‐up^4^	PLCO, 15 years; for all‐cause mortality and 16.9‐year median follow‐up^31^, ^32^	Norrköping, 20‐year follow‐up^47^	Quebec, 8‐year follow‐up^43^
Prostate cancer‐specific mortality rate ratio (RR) between screening and no screening groups	0.80 (95% CI 0.72–0.89)	0.71 (95% CI 0.55–0.91)	0.96 (95% CI 0.85–1.08)	16.9‐year follow‐up (intervention) and 16.7 years (control): 0.93 (95% CI 0.81–1.08)	1.16 (95% CI 0.78–1.73)	Death incidence rate 48.7 and 15.0 for unscreened (control) and screened populations, respectively
All‐cause mortality	0.99 (95% CI 0.97–1.01)	22‐year follow‐up: RR 1.02 (95% CI 0.97–1.07)	10‐year follow‐up: No difference in all‐cause mortality. RR 0.99 (95% CI 0.94–1.03)	15‐year follow‐up: No difference in all‐cause mortality in prostate cancer cases. RR 0.977 (95% CI 0.950–1.004)	Mortality for men with prostate cancer was 69/85 (81%) in the screening group and 252/292 (86%) in the control group	NR
NNI/NND	570/18	22‐year follow‐up: 221/9	NR	NR	NR	NR
Prostate cancer incidence rate ratio (RR) and cumulative incidence (%), where available	1.41 (95% CI 1.36–1.45)	RR = 1.42 (95% CI 1.31–1.53). The cumulative incidence was 18.6% in intervention group and 14.3% in control group. An absolute difference of 4.3% (95% CI 3.1–5.5	4.3% in intervention group and 3.6% in control group RR 1.19 (95% CI 1.14–1.25)	Cumulative incidence: 1.05 (95% CI 1.01–1.09)	5.7% in intervention and 292/7532 3.9% in control group	The incidence rate for screened and unscreened men was 13.7 and 41.6 per 100 000 person years, respectively.
Late‐stage diagnosis; relevant information, including rate ratio (RR), hazard ratio (HR) cumulative incidence and absolute risk reduction, where available	12‐year follow‐up: HR for metastatic disease 0.70 (95% CI 0.60–0.82); 256 cases in screening arm and 410 in control arm. Cumulative incidence of 0.67% and 0.86% per 1000 men for screening and control arms, respectively. Absolute risk reduction of metastatic disease of 3.1 per 1000 men randomised	22‐year follow‐up: Non‐localised tumours: 2.5% in intervention group and 2.8% in control group 18‐year follow‐up: 1.2% diagnosed with advanced disease in intervention group and 1.4% in control group. 1.8% diagnosed with metastatic prostate cancer in intervention group and 2% in control group	0.5% diagnosed as T4, N1 or M1 in intervention group and 0.6% in control group. Group difference 0.91 (95% CI −1.36 to −0.46)	16.9‐year follow‐up: RR for Gleason 8–10: 0.89 (95% CI 0.80–0.99). RR for metastatic disease was 0.85 (95% CI 0.67–1.06)	Advanced tumours (T3–T4, N1 or MX/M1), found in 43.50% (at screening and interval) and in 73.30% of the control group	NR
Low‐grade diagnosis/excess incidence	16‐year follow‐up excess incidence 41%, Finland ERSPC 30%	18‐year follow‐up: 2.6% diagnosed with low‐risk prostate cancer in intervention group and 0.7% in control group	Gleason grade 6 diagnoses: 1.7% in intervention group and 1.1% in control group	16.9‐year follow‐up: RR for Gleason 2–6: 1.17 (95% CI 1.11–1.23). RR for Gleason 7: 1.00 (95% CI 0.93–1.07)	Localised tumours: 56.5% in intervention and 26.7% in control group	NR
Quality of life	15‐year follow‐up Finnish ERSPC, no major quality of life differences between arms. Only statistically significant difference was in ‘urinary bother’, examined using UCLA‐Prostate Cancer Index with score of 77.9 (95% CI 75.2 to 80.5) in intervention compared with 70.9 (95% CI 66.8 to 74.9) in control	NR	NR	NR	NR	NR

*Note*: Key outcomes were sourced from latest search retrieved articles, unless indicated otherwise.In the ERSPC study article a median follow‐up of 15.5 years is quoted with a maximum follow‐up of 16 years reported for multiple centres. This has been simplified in the table to describe median follow‐up as 16 years.

Abbreviations: CAP, Cluster Randomised Trial of PSA Testing for Prostate Cancer; CI, confidence interval; ERSPC, European Randomised Study of Screening for Prostate Cancer; FH, family history; HR, hazard ratio; NND, number needed to diagnose; NNI, number needed to invite; NR, not reported; OR, odds ratio; PLCO, Prostate, Lung, Colorectal and Ovarian Cancer Screening Trial; RR, rate ratio, TNM staging, describes size of tumour (T), detection of cancer cells in lymph nodes (N) and spread of cancer (M).

**TABLE 3:** Summary of prostate cancer outcomes by age, ethnicity and family history, across study trials.

Trial study articles reporting on predefined risk factors	Risk factors		
	Age	Ethnicity	Family history
ERSPC^46^	NR	NR	**General**: Swedish ERSPC at 11.6 years median follow‐up; screening arm had 4932 attendees, 334 of which had positive FH and 4598 had negative FH. **Diagnosis/incidence**: cumulative prostate cancer incidence was 18% for men with a FH vs. 12% for men without (OR 1.6, 95% CI 1.2–2.2). Men with a positive FH had a higher unadjusted risk for overall prostate cancer diagnosis than men with a negative FH (OR 1.61, 95% CI 1.20–2.16). **Biopsy/grade**: no difference in Gleason score and PSA level between men with positive and negative FH. **Mortality**: for men with positive FH compared with men with negative FH OR was (i) 2.97, 95% CI 0.85–10.38, *p* = 0.07 for prostate cancer‐specific mortality and (ii) 1.05, 95% CI 0.75–1.48, *p* = 0.8 for overall mortality **Other analyses**: univariable and multivariable analysis of predicators of prostate cancer incidence, revealed that FH remained an independent predictor for overall (HR of univariate: 1.58, 95% CI 1.21–2.07, *p* = 0.001 but not aggressive (Gleason score of ≥7) prostate cancer (HR for univariate: 1.30, 95% CI 0.82–2.06, *p* = 0.26)
Göteborg^29^, ^45^	**1. General**: 17‐year follow‐up per 1000 person years for screened and unscreened men, aged 50–54 **Diagnosis/incidence**: prostate cancer diagnosis (IRR: 2.56, 95% CI 2.18–3.02) **Biopsy/grade**: prostate cancer metastasis (IRR: 0.43, 95% CI 0.22–0.79) **Mortality**: prostate cancer‐specific mortality (IRR: 0.29, 95% CI 0.11–0.67) and overall mortality (IRR: 0.99, 95% CI 0.89–1.10) **2. General**: 18‐year follow‐up per 1000 person years for screened and unscreened men. **Diagnosis/incidence**: (i) for 50–54 year olds, incidence rate; 1.77 (95% CI 1.54–2.04) (ii) 55–59 year olds; 1.37 (95% CI 1.19–1.59) and for 60–64 year olds; 1.39 (95% CI 1.19–1.62) **Biopsy/grade**: NR **Mortality**: prostate cancer‐specific mortality rate ratio for (i) 50–54 year olds, 0.50 (95% CI 0.20–1.16); (ii) 55–59 year olds, 0.47 (95% CI 0.28–0.79); and (iii) 60–64 year olds; 0.85 (95% CI 0.56–1.28)	NR	NR
CAP^4^	NR	NR	NR
PLCO^33^	NR	NR	**General**: 4833 White men (7.4%) had a family history of prostate cancer at 11.6‐year follow‐up **Diagnosis/incidence**: men with FH had a higher incidence of prostate cancer (16.9% vs. 10.8%) and higher prostate cancer‐specific mortality (0.56% vs. 0.37%). HR 1.47, 95% CI 0.98–2.21. **Biopsy/grade**: NR **Mortality**: screening in men with a positive family history also showed a trend towards decreased prostate cancer‐specific mortality: HR 0.49, 95% CI 0.22–1.1
Norrköping^47^	NR	NR	NR
Quebec^43^	NR	NR	NR

*Note*: Incidence, mortality and grade/stage of diagnosis are indicated (where available), based on latest study trial data from retrieved articles reporting primarily on predefined risk factors.

Abbreviations: CAP, Cluster Randomised Trial of PSA Testing for Prostate Cancer; CI, confidence interval; ERSPC, European Randomised Study of Screening for Prostate Cancer; FH, family history; HR, hazard ratio; IRR, incidence rate ratios; NR, not reported; OR, odds ratio; PLCO, Prostate, Lung, Colorectal and Ovarian Cancer Screening Trial; RR, rate ratio.

The CAP trial reported that after 10 years of follow‐up, a one‐off invitation to undergo PSA screening had no effect on PCa‐specific mortality. The PLCO trial reported that yearly PSA screening did not affect PCa‐specific mortality. However, this trial had similar PSA testing rates in the intervention and control arms during the post‐screening phase—45% in the intervention arm and 45.9% in the control arm. Moreover, in the screening arm biopsy compliance for those with raised PSA was relatively low in PLCO, with an average compliance of 35% (based on four rounds of screening), compared with around 85% for ERSPC (varied between centres) and CAP.^53^, ^54^

The Norrköping trial introduced PSA screening after two rounds of digital rectal examination (DRE) only testing and found that screening did not affect PCa‐specific mortality. The Quebec trial reports that the PSA screening reduces PCa‐specific mortality; however, there are serious concerns of bias with the Quebec trial.

### 3.4 All‐cause mortality

Five trials measured the effect of PSA screening on all‐cause mortality and found no difference in all‐cause mortality between trial arms. However, to our knowledge, only PLCO and CAP pre‐specified all‐cause mortality analysis in their protocol as secondary outcomes.

### 3.5 PCa incidence

Five trials found that men who underwent screening were more likely to be diagnosed with PCa than those in the control arms, who were not screened. In ERSPC, the rate ratio (RR) of PCa diagnosis decreased with follow‐up time, from 1.90 (95% CI 1.83–1.98) after 9 years of follow‐up to 1.41 (95% CI 1.36–1.45) at 16 years of follow‐up. Similarly, the Göteborg trial reported a RR of 1.64 (95% 1.49–1.80) after 14‐year follow‐up, which decreased to 1.42 (95% CI 1.31–1.53) after 22 years of follow‐up, with PLCO trial data also changing from 1.22 (95% CI 1.16 to 1.29) at 10‐year follow‐up to 1.05 (95% CI 1.01–1.09) after 16.9‐year follow‐up. In CAP the RR of being diagnosed with PCa was 1.19 (95% CI 1.14–1.25) after 10 years of follow‐up.

### 3.6 Metastatic PCa incidence

In the ERSPC trial, there was a 30% relative risk reduction in metastatic disease in the intervention arm and an absolute risk reduction of metastatic disease of 3.1 per 1000 randomised men (0.31%) at 12 years of follow‐up. An intention‐to‐screen analysis of the ERSPC Rotterdam arm concluded that, after adjusting for noncompliance and contamination, the risk of metastatic PCa was reduced by 32%.

In the Göteborg trial, the 18‐year follow‐up study reported only a small difference in the incidence of metastatic disease between trial arms, with 1.8% of patients diagnosed with metastatic PCa in the intervention group and 2% in the control group. Similarly, the 22‐year follow‐up to the Göteborg study reported 2.5% and 2.8% of non‐localised tumours (including metastatic disease) in the intervention and control groups respectively (*p* = 0.44). The incidence of metastatic disease alone was not recorded in the 22‐year follow‐up study.

The Norrköping trial's 20‐year follow‐up study identified advanced tumour, defined as the presence of either T3–T4 or MX/M1, in 43.50% of cancers detected in intervention group and 73.30% of cancers detected in control group. However, most of the cancers detected in the screening group were cancers detected at intervention and not at screening. The CAP trial reported no meaningful difference in metastatic disease between trial arms, with 0.5% diagnosed with metastatic disease in the intervention group and 0.6% in the control group. In PLCO, the RR for metastatic disease was 0.85 (95% CI: 0.67–1.06).

### 3.7 Overdiagnosis (excess incidence)

The Göteborg, CAP and PLCO trials reported a higher incidence of low‐grade disease in the screening arms. The ERSPC trial reported a 41% excess incidence of PCa among screened men after 16 years of follow‐up, and the Finnish ERSPC reported a 30% excess incidence. The excess incidence was reduced with longer follow‐up periods.

### 3.8 Quality of life

The impact of PCa screening on quality of life was only reported in the Finnish ERSPC arm. A 5‐ to 15‐year follow‐up analysis compared the disease‐specific and generic quality of life outcomes between men with PCa in the screening and no screening arms and found that, after long‐term follow‐up, men in the screening arm had a slightly higher disease‐specific quality of life scores; but the results were only significant for urinary bother. There were no differences in generic quality of life scores between arms.

### 3.9 Age

Men of different ages were recruited to the six trials examined in this review: 50–64 for Göteborg; 40–80 for Quebec; 55–69 for ERSPC core (data analysis), CAP and Norrköping; and 55–74 for PLCO (Table 1). Göteborg trial articles, found to have low or some concerns of risk of bias, reported the impact of starting screening from a relatively young age on PCa incidence and mortality (Table 3). At 18 years of follow‐up, subgroup analysis revealed that men who start screening at ages 55–59 were more likely to experience a PCa mortality benefit from screening when compared with a cohort of unscreened (control) in the same age range (RR 0.47, 95% CI 0.29–0.78). Also, a 17‐year follow‐up cohort study reported that screened men aged 50–54 had a lower risk of metastatic disease (incidence RR 0.43, 95% CI 0.22–0.79) and death (incidence RR 0.29, 95% CI 0.11, 0.67), than unscreened men (pre‐PSA) from the Malmö Preventive Project.^45^

### 3.10 Family history

The ERSPC and PLCO trials reported on family history outcomes. In both trials, men reporting first‐degree relative(s) diagnosed with PCa were considered to have a positive family history. An analysis of the Swiss ERSPC screening arm at 11.6 years of median follow‐up found that men with a family history were 1.6 times more likely to be diagnosed with PCa than men without a family history (OR 1.6; 95% CI 1.2–2.2). There was no difference in Gleason grade and PSA levels between men with and without family history of PCa. Family history was also not associated with a higher risk of aggressive PCa. Similarly, PLCO trial subanalysis revealed that screened White men with a family history were around 50% less likely to die from PCa, compared with those with a family history who were not screened (HR 0.49, 95% CI 0.22–1.1, *p* = 0.082, on multivariate analysis). Moreover, the median time to death for screened men was found to be slightly higher (3527 days) than for control (3370 days). However, this analysis was not found to be clinically significant.

### 3.11 Ethnicity

No studies assessed the harms and benefits of screening by ethnic groups. The PLCO trial was the only study that reported ethnicity data among those included, which highlights the underrepresentation of ethnic minority men in RCTs and cohort studies, resulting in a lack of statistical power to measure and assess clinical endpoints and quality of life. The proportion of Black men enrolled in the PLCO study was no more than 4.6%.^31^

## 4 DISCUSSION

In this review, we found that repeated PSA screening reduces the risk of PCa‐specific mortality and metastatic disease but as expected, has no effect on all‐cause mortality, likely because the number of patients needed to detect this difference is too high and trials are not powered to detect a difference in that endpoint. The benefit of screening on PCa‐specific mortality and metastatic disease increases with follow‐up time. After 13 years of median follow‐up, the ERSPC trial reported a 21% relative risk reduction in PCa‐specific mortality. These results align with the Göteborg trial, which reported a 30% relative risk reduction after 22 years of follow‐up. Repeated screening also significantly reduces the risk of metastatic disease by between (i) 15% in the Göteborg trial and after almost 17 years of follow‐up and (ii) 30% in ERSPC after 12 years of follow‐up. In contrast, evidence from the CAP trial showed there was little to no effect in PCa‐specific mortality, but a significant increase in the detection of low‐grade cancers. This may be due to the length of follow‐up, with benefits of screening previously shown to increase in ERSPC trial at longer follow‐up periods. Furthermore, the CAP trial only included a single round of screening with a PSA testing compliance rate of only 36% in the intervention arm, compared with compliance rate of ≥70% across multiple screening rounds in the Norrköping study, 82% in ERSPC and 85% for PLCO trial.^35^, ^42^, ^47^, ^54^

Although repeated PSA screening leads to a reduction in PCa‐specific mortality, the NNI and NND to prevent one PCa death remains high with 570 and 221 needed to be invited to screening and 18 and nine diagnosed to prevent one PCa death in ERSPC and the Göteborg trial, respectively. Screening also results in an excess incidence of PCa and an increase in diagnosis of low‐grade disease. This leads to serious concerns about the overdiagnosis of clinically insignificant PCas that would not have caused any harm, subsequent overtreatment and physical and psychological harms associated with the diagnostic process. Although recent studies suggest that pre‐biopsy MRI might reduce the harms associated with overdiagnosis^55^ to our knowledge, no PCa screening RCTs have reported the impact of pre‐biopsy MRI on PCa‐specific mortality and metastatic disease after long‐term follow‐up. The effect of screening on PCa mortality and metastatic disease varied in different trials. Some showed no effect, while others demonstrated a significant reduction. Factors such as protocol variations, randomisation techniques, screening intensity and duration could account for these differences. Additionally, compliance levels and contamination rates may also have influenced the observed outcomes.

It has been suggested that noncompliance and contamination dilute the effect of screening.^56^ In the PLCO trial, 40% of men in the control arm had at least one PSA on the first screening round, this increased to 52% by the sixth screening round. Eighty‐five per cent men assigned to the intervention arm were compliant with the PSA screening protocol.^35^ Interestingly, one article has cited that rate of contamination in PLCO control arm could be as high as 90%, making interpreting results difficult.^57^ In the Göteborg trial, 72% men in the control group reported having at least one PSA test during the study period, when compared with 87% of men in the screening group.^24^ In the Finnish arm of ERSPC, 62.7% of men had at least one PSA test in the control arm and 85.2% in the screening arm at 12‐year follow‐up.^30^ PSA screening trials are commonly referred to as a comparison between organised PSA screening and opportunistic PSA screening due to the significant number of men, in the United States and Europe, accessing PSA tests in the control group.^38^ An analysis of the Göteborg trial after 18 years of follow‐up found that opportunistic PSA screening had little if any effect on PCa‐specific mortality and was associated with greater overdiagnosis in comparison with organised screening. In opportunistic screening, the number of men needed to be invited to prevent one PCa death is almost twice of that in those enrolled in organised screening programme every 2 years.^38^

Several studies have tried to adjust for contamination and noncompliance to measure the ‘true’ effect of organised PSA screening in ERSPC. An analysis of the ERSPC Rotterdam arm at 13 years median follow‐up found that after correcting for contamination and noncompliance, the relative risk of PCa‐specific mortality is reduced. More specifically, this reduction in PCa‐specific mortality after screening rose from 32% to 51%.^56^ Similarly, another intention‐to‐screen analysis of the ERSPC Rotterdam arm, based on an average follow‐up of 8.9 years, found that after three rounds of four‐yearly PSA screening, the reduction in risk of metastatic PCa rose from 25% to 32% after adjusting for noncompliance and contamination.^28^ However, these corrections have the potential to bias results and reduce internal validity of study.

We did not find good quality evidence of the harms and benefits of screening by family history, ethnicity or age; although European Guidelines have been made based on age.^12^ For example, one study with high risk of bias reported that screening White men with a family history reduces the risk of PCa‐specific mortality by 50%, compared with those with a family history who were not screened.^33^ However, the lack of prospective control arms, for example, men not screened with and without family history, means definitive conclusion cannot be drawn. Furthermore, a recent UK Genetic Prostate Cancer study examined a cohort of diagnosed men with a first, second or third degree relative with PCa and found that family history was inversely associated with a higher risk of overall and PCa‐specific mortality.^17^ Interestingly, the authors proposed that the reduction in mortality may be due to ‘awareness’ of risk, as there was no reduction in mortality observed when patients were diagnosed with PCa before their relatives.^17^ Further research is necessary to determine the excess incidence of low‐risk PCa in men with a family history who undergo PCa screening compared with those who do not.

In this review, the only RCT that assessed the effect of screening by age was the Göteborg trial. The 22‐year follow‐up to the Göteborg study, found to have low risk of bias, reported that men were more likely to experience mortality benefit when they were screened before the age of 60, while those screened at 60 years of age or above were found to be diagnosed with more advanced PCa.^40^ Similarly, an article reporting on the 18‐year follow‐up of the Göteborg study and examining the effect of sociodemographic variables on outcomes reported that men who started screening at 55–59 years of age were more likely to acquire a PCa‐specific mortality benefit and that screening men in their early 50s led to a lower risk of metastatic disease.^29^ This finding was also further supported by the latest 24‐year follow‐up Göteborg study, which found that screening men between the ages of 50–55 meant that these screened men had halved the risk of a PCa diagnosis, compared with men first screened at 60 years of age. Men who started screening between the ages of 50–55 were also found to have an NNI and NND in the range of 189–202 and 8–9, respectively. While for men who started screening at 60, NNI and NND were 416 and 18, respectively.^15^ However, as this subgroup analysis was not conducted at shorter follow‐up periods, it is not clear how key values such as NNI and NND would have differed; one possibility is that values would have been higher (Table S6). More broadly, the relevance of trial results, from long follow‐up periods to men with different life expectancies and with different comorbidities remains unclear, as older men may be more likely to die from comorbidities than PCa.

Moreover, the benefits observed in the ERSPC multi‐trial arm study were only applicable to men aged between 50 and 69 years old, with no evidence showing a mortality benefit for those under age 50 or over age 69. An intention‐to‐treat analysis, conducted on the entire ERSPC Rotterdam cohort (ages 50–74), also showed no statistically significant difference in PCa‐specific mortality among men who were randomised at age 70 or older after a follow‐up period of 21 years.^58^ Furthermore, a recent article has highlighted that PSA testing in men over 70 was associated with 40% of overdiagnoses and suggested that to reduce the harms of overdiagnosis and overtreatment, a screening programme implemented in the form of a comprehensive risk‐adapted early detection programme would likely need to restrict PSA testing to men aged 50–70.^8^ However, reports from the main multi‐arm ERSPC study lacked contextual information on the differences in life expectancy across the different participating countries, with differences in demographics and health characteristics likely to play a role, including factors such as average age of population, ethnicity, rates of obesity and the use of different healthcare models which impact population access to healthcare and policy guidance.

None of the six clinical trials included in the analysis examined the harms and benefits of PSA screening in Black men. Furthermore, the ERSPC and CAP trials lacked an ethnicity breakdown, while only around 4% of the cohort included in PLCO were Black men, despite Black population as a whole accounting for around 12% to 13% of the general US population during trial study period. The PLCO study was also not powered to detect differences in outcomes such as PCa‐specific mortality for Black men. Aside from the lack of power, contamination issues in PLCO trial also further hindered accurate generalisability of study results by ethnicity.

Future studies should consider implementing recruitment strategies to intentionally overrepresent Black men to adequately assess specific outcomes in this population and prevent issues related to insufficient sample size or power. The Imperial Prostate 1 Prostate Cancer Screening Trial Using Imaging (IP‐1 PROSTAGRAM study), for example, implemented a direct‐to‐community recruitment strategy to increase the representation of ethnic minority groups in the trial, which resulted in a cohort that included 32.4% Black, 23% Asian and 6.6% other or mixed‐race men aged 50 to 69 years.^59^

Our research supports previous analyses that found inconclusive evidence on the harms and benefits of PSA PCa screening by risk factors.^6^, ^7^ Our review also supports the findings of other reviews which indicated that screening reduces PCa mortality rates and metastatic occurrences but increases the incidence of low‐grade and overall PCa.^54^

Moreover, a treatment trial linked to CAP called ProtecT showed participants diagnosed with localised cancer were likely to survive over a 15‐year period regardless of treatment intervention, with the highest prostate‐specific mortality observed for those assigned to active surveillance, affecting only 3.1% of patients.^60^ However, more research is required to determine the long‐term physical and psychological consequences of undergoing PCa screening on high‐risk men.

In conclusion, we found a lack of strong evidence on the harms and benefits of PSA screening by risk factors. This contrasts with the current practice approach of shared decision‐making, which relies on information about patients' risk factors to make informed decisions about the PSA test. Further research is needed to understand how ethnicity and family history impact the effectiveness of screening programmes and to better assess the long‐term harms and benefits of PSA testing, particularly in high‐risk populations, in the modern diagnostic setting and since the introduction of pre‐biopsy MRI.

## AUTHOR CONTRIBUTIONS

All named authors contributed to the creation of this mauscript.

## CONFLICT OF INTEREST STATEMENT

There were no conflicts of interest declared by any of the authors who contributed to the creation of this review.

## Supporting information


**Table S1.** Study protocol and inclusion/exclusion criteria.
**Table S2.** Search terms and combinations used in PubMed. The following selection filters were applied: English language, clinical trials and randomised controlled trial studies published between 01/01/1990 and 25/01/2023.
**Table S3.** Search terms and combinations used in Cochrane Central Register of Controlled Trials. The following selection filters were applied: English language, clinical trials and randomised controlled trial studies published between 01/01/1990 and 25/01/2023.
**Table S4.** Strategy, search terms and combinations used to retrieve relevant grey literature and clinical trials.
**Table S5.** Domains and adaptations for quality assessment tools.
**Table S6.** Key outcomes across studies that cite multiple follow‐up years. Where possible, outcome measures were sourced from original study articles reporting on specific follow‐up years. However, it is important to note that in some instances, the latest trial follow‐up reports quote different values for measures previously reported on older follow‐up study articles. For clarity, measures reported from the latest ERSPC (16‐year) and Göteborg (22‐year) trial study articles are shown in italics. These measures were used in the main results section of this paper.
