# Supplementary material for: Variation in harms and benefits of prostate‐specific antigen screening for prostate cancer by socio‐clinical risk factors: A rapid review
Source: BJUI Compass. 2024 Feb 9;5(5):417–32. doi: 10.1002/bco2.326 (PMC11090766; doi:10.1002/bco2.326)
Supplement: Supplementary file 1 — Table S1. Study protocol and inclusion/exclusion criteria. Table S2. Search terms and combinations used in PubMed. The following selection filters were applied: English language, clinical trials and randomised controlled trial studies published between 01/01/1990 and 25/01/2023. Table S3. Search terms and combinations used in Cochrane Central Register of Controlled Trials. The following selection filters were applied: English language, clinical trials and randomised controlled trial studies published between 01/01/1990 and 25/01/2023. Table S4. Strategy, search terms and combinations used to retrieve relevant grey literature and clinical trials. Table S5. Domains and adaptations for quality assessment tools. Table S6. Key outcomes across studies that cite multiple follow‐up years. Where possible, outcome measures were sourced from original study articles reporting on specific follow‐up years. However, it is important to note that in some instances, the latest trial follow‐up reports quote different values for measures previously reported on older follow‐up study articles. For clarity, measures reported from the latest ERSPC (16‐year) and Göteborg (22‐year) trial study articles are shown in italics. These measures were used in the main results section of this paper. [file BCO2-5-417-s001.docx]

**Supplementary Tables**

***Supplementary Table 1.*** *Study protocol and inclusion/exclusion criteria*

| **Study Protocol** | **Details** | |
| --- | --- | --- |
| **Review Question** | What are the benefits and harms of PSA based prostate cancer screening and the diagnostic pathway? Do the benefits and harms vary by risk factors (age/ethnicity/family history)? | |
| **Type of study to be reviewed** | Randomised controlled trials and cohort studies | |
| **Method of review** | Rapid review | |
| **Main condition of interest** | Prostate cancer | |
| **Aim of study** | To analyse i) the latest evidence on the relative harms and benefits of screening and diagnostic pathways and ii) identify how this applies to men at risk and higher risk of prostate cancer | |
| **Date** | 01/01/1990-25/01/2023 | |
| **Language** | English | |
| **Country** | No restriction by country | |
| **Information sources and databases** | - PubMed and Cochrane Central Register of Controlled Trials via Wiley. - Limited search of websites and references including from relevant guidelines, clinical trial search portals as well as from key trial studies | |
| **Screening process** | A blinded abstract and full-text screening process will be conducted by two reviewers using Rayyan systematic review tool, which contains features such as i) autodeletion and deduplication of uploaded articles ii) the ability to conduct a blinded review process through separate login systems for individual reviewers iii) and the ability to manually screen articles and later automate process for remaining articles. Any conflict between reviewers will be reconciled by a third team member. | |
| **Study records and assignment of articles for data extraction** | Rayyan will be used for study selection and Zotero reference system for storing references, with article sharing between software enabled via BibTeX format. Zotero will be used to generate ‘keys’ (codes) and facilitate the random assignment of articles to independent reviewers ahead of data extraction. | |
| **Data extraction** | A data extraction form will be developed and tested in Excel using information from a variety of relevant sources such as main study report and supplementary papers. Data extraction will focus on general study information, methods, number of participants, intervention used, outcomes assessed and other items such as funding sources and study conclusions | |
| **Risk of bias assessment tools** | Randomised controlled trials and cohort studies will be analysed using relevant Cochrane risk of bias assessment tools | |
| **Review team details** | - Abel Tesfai: [abel.tesfai@prostatecanceruk.org](mailto:abel.tesfai@prostatecanceruk.org) - Natalia Norori: [natalia.norori@prostatecanceruk.org](mailto:natalia.norori@prostatecanceruk.org) - Thomas A Harding: [tom.harding@bristol.ac.uk](mailto:tom.harding@bristol.ac.uk) - Yui Hang Wong: [harris.wong@prostatecanceruk.org](mailto:harris.wong@prostatecanceruk.org) - Matthew David Hobbs: [matthew.hobbs@prostatecanceruk.org](mailto:matthew.hobbs@prostatecanceruk.org) | |
| **Review team organisational affiliation** | Prostate Cancer UK | |
| **Population, Intervention, Comparator and Outcome (PICO)** | **Inclusion criteria** | **Exclusion criteria** |
| **Population** | Men over 45, Men with a first-degree relative with prostate, breast, ovarian cancer, Black men. | Men who i) have been previously diagnosed with prostate cancer and/or are under the age of 45. |
| **Intervention(s)** | PSA based screening with or without MRI | Non-PSA based screening methods |
| **Comparator(s)** | Current standard of care (no organised screening) | - |
| **Outcome(s)** | - Outcomes focused on following benefits of screening i) reduction in prostate cancer-specific mortality ii) reduction in all-cause mortality iii) reduction in the iii) incidence of aggressive or /metastatic disease iv) improvement in quality of life, and v) quality of life/psychological effects. - Outcomes also focused on the following harms associated with screening namely i): overtreatment ii) and treatment specific harmful effects iii) overdiagnosis iv) complications arising from use of biopsy, including urinary tract infection, sepsis and general infection as well as v) psychological effects | |

***Supplementary Table 2.*** *Search terms and combinations used in PubMed.* The following selection filters were applied: English language, clinical trials and randomised controlled trial studies published between 01/01/1990 and 25/01/2023.

| #1 | Prostatic Neoplasms OR Prostate Cancer |
| --- | --- |
| #2 | Middle Aged OR Aged |
| #3 | Ethnicity OR Age Factors OR Risk Factor* |
| #4 | Medical History Taking OR Family History |
| #5 | Ovarian Neoplasms / Diagnosis* |
| #6 | Breast Neoplasms / Diagnosis* |
| #7 | PSA OR Prostate-Specific Antigen / Blood* |
| #8 | #1 AND #7 AND (#2 OR #3 OR #4 OR #5 OR #6) |
| #9 | Magnetic Resonance Imaging* OR Prostatic Neoplasms/Diagnostic Imaging* OR Prostate / Diagnostic Imaging* |
| #10 | Magnetic Resonance Imaging / Methods |
| #11 | Early Detection Of Cancer / Methods OR Early Detection Cancer* / Economics OR Prostate / Diagnostic Imaging |
| #12 | PSA OR Prostate-Specific Antigen / Blood* |
| #13 | #12 AND (#9 OR #10 OR #11) |
| #14 | Referral Guidance OR Diagnostic Pathway OR Pathway OR Suspected Cancer Pathway OR Standard Of Care OR Quality Indicators, Health Care OR Practice Patterns, Physicians' |
| #15 | Mass Screening / Methods* OR Early Detection Of Cancer |
| #16 | Image-Guided Biopsy / Methods |
| #17 | #14 OR #15 OR #16 |
| #18 | Outcome Assessment, Health Care OR Benefits OR Benefits And Costs OR Harms OR Patient Harm OR Mortality OR Prostate Cancer-Specific Survival OR Prostate Cancer-Specific Death |
| #19 | Overdiagnosis OR Overtreatment |
| #20 | Quality Of Life OR Emotions OR Decision Making |
| #21 | #18 OR #19 OR #20 |
| #22 | Drugs OR Recurrence OR Active Surveillance OR Hormone |
| #23 | #8 AND #13 AND #17 AND #21 NOT #22 |

***Supplementary Table 3.*** *Search terms and combinations used in Cochrane Central Register of Controlled Trials.* The following selection filters were applied: English language, clinical trials and randomised controlled trial studies published between 01/01/1990 and 25/01/2023.

| #1 | Prostatic neoplasms OR Prostate cancer |
| --- | --- |
| #2 | Middle Aged OR Aged |
| #3 | Ethnicity OR Age Factors OR Risk Factor* OR Medical History Taking OR Family History |
| #4 | Ovarian Neoplasms diagnosis OR Breast Neoplasms diagnosis |
| #5 | PSA OR Prostate-Specific Antigen |
| #6 | #1 AND #5 (#2 OR #3 OR #4) |
| #7 | Magnetic Resonance Imaging* OR Prostatic Neoplasms diagnostic Imaging OR Prostate diagnostic imaging |
| #8 | Magnetic Resonance Imaging methods OR Early Detection of Cancer methods OR Prostate diagnostic imaging |
| #9 | PSA OR Prostate-Specific Antigen |
| #10 | #9 AND (#7 OR #8) |
| #11 | Referral guidance OR Diagnostic pathway OR Pathway OR Suspected Cancer Pathway OR Standard of Care OR Quality Indicators, Health Care OR Practice Patterns, Physicians' |
| #12 | Mass Screening methods* OR Early Detection of Cancer OR Image-Guided Biopsy |
| #13 | #11 OR #12 |
| #14 | Outcome Assessment, Health Care OR Benefits OR Benefits And Costs OR Harms OR patient harm OR mortality OR prostate cancer-specific survival OR prostate cancer-specific death |
| #15 | Overdiagnosis OR Overtreatment OR Quality of Life OR Emotions OR Decision Making |
| #16 | #14 OR #15 |
| #17 | Drugs OR Recurrence OR Active Surveillance OR Hormone |
| #18 | #6 AND #10 AND #13 AND #16 NOT #17 |

***Supplementary Table 4.*** *Strategy, search terms and combinations used to retrieve relevant grey literature and clinical trials.*

| Source | Search strategy/terms |
| --- | --- |
| Grey literature | Relevant national and international guidelines were analysed by the review team, specifically the European Association of Urology’s chapter on prostate cancer diagnostic evaluation <https://uroweb.org/guidelines/prostate-cancer> |
| Clinical trial information from search portals | 1) BioMed Central; selected ‘Prostate Cancer’ option 2) Cochrane; searched 'prostate cancer screening', 3) ClinicalTrials.gov; searched 'prostate cancer and cancer screening' and 4) WHO International Clinical Trials Registry Platform; 'prostate cancer and screening' |

***Supplementary Table 5****. Domains and adaptations for quality assessment tools.*

|  | **RoB 2** | **RoB 2 for Cluster randomised trials** | **Robins-I** |
| --- | --- | --- | --- |
| Domains | - domain 1; risk of bias arising from the randomisation process - domain 2; risk of bias due to deviations from the intended interventions (effect of assignment to intervention) - domain 3; risk of bias due to missing outcome data - domain 4; risk of bias in the measurement of the outcome - domain 5; risk of bias in the selection of the reported result. | - domain 1; risk of bias arising from the randomisation process and risk of bias arising from the timing of identification or recruitment of participants in a cluster-randomised trial - domain 2; risk of bias due to deviations from the intended interventions (effect of assignment to intervention) - domain 3; risk of bias due to missing outcome data - domain 4; risk of bias in the measurement of the outcome - domain 5; risk of bias in the selection of the reported result. | - domain 1; bias due to confounding - domain 2; bias in the selection of participants into the study - domain 3; bias in classification of interventions - domain 4; bias due to deviations from intended interventions - domain 5; bias due to missing data - domain 6; bias in the measurement of outcomes - domain 7; bias in the selection of the reported result |
| Adaptations | N/A | N/A | Judgments were consolidated to reflect the lack of critical risk of bias found in studies |

**Supplementary Table 6. Key outcomes across studies that cite multiple follow-up years**. Where possible, outcome measures were sourced from original study articles reporting on specific follow-up years. However, it is important to note that in some instances, the latest trial follow-up reports quote different values for measures previously reported on older follow-up study articles. For clarity, measures reported from the latest ERSPC (16-year) and Göteborg (22-year) trial study articles are shown in italics. These measures were used in the main results section of this paper.

| Median follow-up and outcome measures | ERSPC (core) | Göteborg | PLCO |
| --- | --- | --- | --- |
| Median follow-up times (years) | 16 (Hugosson, J et al., 2019)  13 (Schröder, FH et al., 2014)  11 (Schröder, FH et al., 2012)  9 (Schröder, FH et al., 2009) | 22 (Frånlund,M et al., 2022)  18 (Hugosson, J et al., 2018)  14 (Hugosson, J et al., 2010) | 16.9 (Pinsky, PF et al.,2018)  15 (Pinsky, PF et al., 2017)  13 (Andriole, GL., 2012)  10 (Andriole, GL., 2009) |
| Prostate cancer-specific mortality rate ratio (RR) in screening and control groups | 16-year follow-up: 0.80 (95% CI 0.72–0.89)  13-year follow-up: 0·79 (95% CI 0·69 to 0·91)  *13-year follow-up (according to the latest 16-year study article):* 0.79 (95% CI 0.69–0.90)  11-year follow-up: 0.79 (95% CI 0.67 to 0.92)  *11-year follow-up (according to the latest 16-year study article): 0.78 (*95% CI *0.67–0.91)*  9-year follow-up: 0.85 (95% CI 0.73–1.00)  *9-year follow-up (according to the latest 16-year study article): 0.84 (*95% CI *0.70–1.00)* | 22-year follow-up: 0.71 (95% CI 0.55–0.91)  18-year median follow-up:  0.65 (95% CI 0.49–0.87)  *18-year follow-up (according to the latest 22--year study article): 0.65* (95% CI 0.48 – 0.88)  14-year median follow-up: 0.56 (95% CI 0.39– 0.82)  *14-year follow-up (according to the latest 22-year study article): 0.55* (95% CI 0.37 – 0.82) | 16.9-year follow-up (intervention) and 16.7 years (control): 0.93 (95% CI 0.81–1.08)  15-year follow-up: reported for 0-12 years as 1.003 (95% CI 0.81-1.25)  13-year follow-up: 1.09 (95% CI 0.87- 1.36)  10-year follow-up: 1.13 (95% CI 0.75 to 1.70) |
| All-cause mortality rate ratio (RR) | 16-year follow-up for core age group 55-6: 0.99 (95% CI 0.97 - 1.01)  13-year follow-up: 1.00 (95% CI 0.98–1.02)  11-year follow-up: 0.99 (95% CI 0.97-1.01  9-year follow-up: 0.99 (95% CI 0.97-1.02) | 22-year follow-up: 1.02 (95% CI 0.97-1.07)  18-year follow-up: 0.99  (95% CI 0.94–1.05)  *18-year follow-up (according to the latest 22--year study article): 1.00 (95% CI 0.95-1.06)*  14-year follow-up: no difference  *14-year follow-up (according to the latest 22--year study article): 1.01 (95% CI 0.94-1.07)* | 16.9-year follow-up: NR  15-year follow-up: 0.977 (95% CI 0.950–1.004)  13-year follow-up: no difference  10-year follow-up: no difference |
| NNI / NND | 16-year follow-up: 570 / 18  13-year follow-up: 781/27  *13-year follow-up (according to the latest 16-year study article):742/26*  11-year follow-up: 936/33  *11-year follow-up (according to the latest 16-year study article): 962/34*  9-year follow-up: this article displays the number needed to screen/number needed to treat as 1410/48  *9-year follow-up (according to the latest 16-year study article): 1947/76* | 22-year follow-up: 221/9, according to the main text in the study article. However, ‘217’ was reported as NNI in the article's abstract  18-year follow-up: 231/10  *18-year follow-up (according to the latest 22--year study article): 243/11*  14-year median follow-up: 293/12  *14-year follow-up (according to the latest 22--year study article): 293/13* | NR |
| Prostate cancer incidence rate ratio (RR) and/or cumulative incidence (%) | 16-year follow-up: 1.41 (95% CI 1.36–1.45)  13-year follow-up: 1·57 (95% CI 1·51–1·62)  *13-year follow-up (according to the latest 16-year study article): 1.54 (*95% CI *1.49–1.59)*  11-year follow-up: 1.68 (95% CI 1.62 to 1.75)  *11-year follow-up (according to the latest 16-year study article): 1.65 (95% CI 1.59–1.71)*  9-year follow-up: 1.90 (95% CI 1.83-1.98) according to 16-year follow-up paper (NR in the original paper).  The cumulative incidence of prostate cancer was 8.2%  in the screening group and 4.8% in the control group  *9-year follow-up (according to the latest 16-year study article): 1.90 (95% CI 1.83–1.98)* | 22-year follow-up: RR = 1.42 (95% CI 1.31‐1.53)  The cumulative incidence was 18.6% in the intervention group and 14.3% in the control group.  An absolute difference of 4.3% (95% CI 3.1–5.5  18-year median follow-up: 1.51 (95% CI 1.39–1.64)  *18-year follow-up (according to the latest 22--year study article): 1.51 (95% CI 1.39–1.64)*  14-year median follow-up: 1.63 (95% CI 1.49–1.80)  *14-year follow-up (according to the latest 22--year study article): 1.64 (95% CI 1.49-1.80)* | 16.9 years follow-up: cumulative incidence: 1.05 (95% CI 1.01–1.09)  15 years follow-up: NR  13 years follow-up: 1.12 (95% CI 1.07-1.17)  10 years follow-up: 1.22 (95% CI 1.16 to 1.29) |

Abbreviations: CAP, Cluster Randomised Trial of PSA Testing for Prostate Cancer; CI, confidence interval; ERSPC, European Randomised Study of Screening for Prostate Cancer; FH, family history; HR, hazard ratio; NND, number needed to diagnose; NNI, number needed to invite; NR, not reported; OR, odds ratio; PLCO, Prostate, Lung, Colorectal and Ovarian cancer screening trial; RR, rate ratio.
